# Forward Period Analysis Method of the Periodic Hamiltonian System

**DOI:** 10.1371/journal.pone.0163303

**Published:** 2016-10-11

**Authors:** Pengfei Wang

**Affiliations:** 1 State Key Laboratory of Numerical Modeling for Atmospheric Sciences and Geophysical Fluid Dynamics (LASG), Institute of Atmospheric Physics, Chinese Academy of Sciences, Beijing, China; 2 Center for Monsoon System Research, Institute of Atmospheric Physics (CMSR), Chinese Academy of Sciences, Beijing, China; Shanxi University, CHINA

## Abstract

Using the forward period analysis (FPA), we obtain the period of a Morse oscillator and mathematical pendulum system, with the accuracy of 100 significant digits. From these results, the long-term [0, 10^60^] (time unit) solutions, ranging from the Planck time to the age of the universe, are computed reliably and quickly with a parallel multiple-precision Taylor series (PMT) scheme. The application of FPA to periodic systems can greatly reduce the computation time of long-term reliable simulations. This scheme provides an efficient way to generate reference solutions, against which long-term simulations using other schemes can be tested.

## Introduction

The Hamiltonian system plays a vital part in describing the evolution of a physical system. Various numerical schemes, including the Euler, Runge–Kutta, linear multistep methods, and some low-order Taylor methods, have been designed to describe the dynamical system. However, these methods are non-structure-preserving. In addition, they lead to unstable computation or incorrect solutions. The symplectic method [1–3], a common structure-preserving method, conserves the area or volume of the system during computation. The square conservation also preserves the structure through conserving the length of the simulated system [4]. These structure-preserving methods allow the Hamiltonian constant to remain constant, or only vary periodically during the entire integration time range [0,*t*]. The structure-preserving methods have the advantages in that they provide a true long-term trajectory of the simulated system and stabilize the computation process.

However, these structure-preserving methods need to be improved. First, when tackling the nonlinear Hamiltonian systems, some of the symplectic methods strongly depend on the generating function. These implicit methods are applied to solve nonlinear algebraic equations at each step, and thus efficiency becomes a problem. Some symplectic methods are based on the Runge–Kutta method, the order of which is generally less than 10 (rarely more than 15), to avoid a complicated procedure. Some other high-order explicit symplectic methods are used to study separable Hamiltonian systems [2,5], but these explicit methods usually have an order of less than 10, and are limited to separable Hamiltonian systems.

Second, although symplectic methods have the advantage of choosing a larger step size than in a classical approach and thus saving simulation time, such an increase in step-size causes large errors in the primary variables. This occurs despite that there is no modification in the trajectory structure. Gladman [6] has said that “…the conservation of the integrals is not a problem for the symplectic integration algorithms (SIAs) but the phase errors can still be uncomfortable after a large number of orbits. If one wanted to integrate the Earth for the lifetime of the solar system it is doubtful that these two SIAs could perform the ~10^9^ orbit integration reliably. This is not necessarily too disheartening, since no other integration scheme known to the authors could perform the integration either.”

The phase trajectory of a Hamiltonian system is one of the most basic requirements. However, given the period of the Hamiltonian system, the symplectic method provides no special insight, and only gives approximate numerical periods with the precision proportional to the order of method and step-size. The long-term integration of a dynamical system is a challenge but urgent task in many fields. Orders of magnitude of time periods in physics range from the Planck time (5.39 × 10^−44^ s) to the age of universe (about 10^17^ s). Thus, a meaningful non-dimensional time for a dynamical system is within 10^60^ orders of magnitude. Simulation of the position of dynamical variables (not the trajectory structure) in the ultra long-term (i.e., *t* = 10^60^) is still a time consuming task, even for a periodic dynamical system. The purpose of this study is to provide a reliable scheme to solve the issues mentioned above.

## Materials and Methods

The parallel multiple-precision Taylor (PMT) method [7–12] is originally designed to solve nonlinear chaotic systems. It provides ultra-high reliable solutions for longer times than other methods. The order of the PMT method can be very high compared to other traditional approaches. Here, the application of the PMT method to a nonlinear Hamiltonian system is examined. The orbits of the system for two atoms with Morse potential energy [13] are

$$\left\{ \begin{array}{l} \frac{dx}{dt}=p\\ \frac{dp}{dt}=e^{-2x}-e^{-x} \end{array}\right.\;and\;H=\frac{p^{2}}{2}+\frac{1}{2}\left(e^{-2x}-2e^{-x}\right).$$ (1)

The initial values are *x*_0_ = 0 and $p_{0}=\sqrt{1-0.02}$, where *x* is the displacement and *p* is the momentum of the particles. Using the substitution *y* = *e*^−*x*^ yields

$$\left\{ \begin{array}{l} \frac{dy}{dt}=-py\\ \frac{dp}{dt}=y^{2}-y \end{array}\right.,$$ (2)

where *y*_0_ = 1 and $p_{0}=\sqrt{1-0.02}$, and $H=\frac{p^{2}}{2}+\frac{1}{2}\left(y^{2}-2y\right)$ is a constant.

The Taylor series expansions relevant to solving (2) are

$$\left\{ \begin{array}{l} y_{n+1}=y_{n}+\sum_{k=1}^{M}\alpha_{k}h^{k}\\ p_{n+1}=p_{n}+\sum_{k=1}^{M}\beta_{k}h^{k} \end{array}\right.,$$ (3)

where the coefficients are given by $\alpha_{k}=\frac{1}{k!}\frac{{\text{d}}^{k}y\left(t_{n}\right)}{\text{d}t^{k}}$ and $\beta_{k}=\frac{1}{k!}\frac{{\text{d}}^{k}p\left(t_{n}\right)}{\text{d}t^{k}}$; and *h* is the step-size. The coefficients are determined from the initial conditions $\left\{ \begin{array}{l} \alpha_{0}=y_{n}\\ \beta_{0}=p_{n} \end{array}\right.$, and the relations

$$\left\{ \begin{array}{l} \alpha_{k+1}=\frac{-1}{k+1}\left(\sum_{i=0}^{k}\alpha_{k-i}\beta_{i}\right)\\ \beta_{k+1}=\frac{1}{k+1}\left(\sum_{i=0}^{k}\alpha_{k-i}\alpha_{i}-\alpha_{k}\right) \end{array}\right..$$ (4)

More details associated with the parallel scheme are referred to Wang et al. [7]. The solution of (1) may be expressed as *x*_*n*+1_ = −ln *y*_*n*+1_, after obtaining the values of *y*_*n*+1_ and *p*_*n*+1_. If the order, *M*, is larger than 100, the parallel scheme greatly reduces the computation time; if *M* is smaller than 30, one CPU is generally sufficient to perform the calculation on a reasonable timescale.

The symplectic method used to solve (1) is a 2^nd^ order explicit method (SE2), as discussed by Qin et al. [14]:

$$u_{1}=p_{k}-hc_{1}f\left(x_{k}\right),v_{1}=x_{k}+hd_{1}g\left(u_{1}\right),$$

$$p_{k+1}=u_{1}-hc_{2}f\left(v_{1}\right),x_{k+1}=v_{1}+hd_{2}g\left(p_{k+1}\right),$$

where *c*_1_ = 0, *c*_2_ = 1, $d_{1}=d_{2}=\frac{1}{2}$, *f*(*x*) = −(*e*^−2*x*^ − *e*^−*x*^), and *g*(*p*) = *p*.

The second and more complex example is a non-separable Hamiltonian system, which is often broadly defined as:

$$\left\{ \begin{array}{l} \frac{dp}{dt}=p\sin q\\ \frac{dq}{dt}=p+\cos q \end{array}\right..$$ (5)

The Hamiltonian is $H=\frac{1}{2}p^{2}+p\cos q$, and the initial values are *q*_0_ = 0, *p*_0_ = 1:

$$\left\{ \begin{array}{l} p_{n+1}=p_{n}+\sum_{k=1}^{M}\alpha_{k}h^{k}\\ q_{n+1}=q_{n}+\sum_{k=1}^{M}\beta_{k}h^{k} \end{array}\right.,$$

where *b*(*t*) = sin *q*, *g*(*t*) = cos *q*, *c*(*t*) = *p* sin *q*, *d*(*t*) = *p* + cos *q* and the *k*^th^ Taylor coefficients are *b*_*k*_, *g*_*k*_, *c*_*k*_, and *d*_*k*_. Therefore, other coefficients are: $\alpha_{m+1}=\frac{1}{m+1}c_{m}$, $\beta_{m+1}=\frac{1}{m+1}d_{m}$, $b_{m}=\frac{1}{m}\sum_{i=1}^{m}ig_{m-i}\beta_{i}$, $g_{m}=\frac{-1}{m}\sum_{i=1}^{m}ib_{m-i}\beta_{i}$, $c_{m}=\sum_{i=0}^{m}\alpha_{m-i}b_{i}$, *d*_*m*_ = *α*_*m*_ + *g*_*m*_, and the initial coefficients are *α*_0_ = *p*_0_, *β*_0_ = *q*_0_, *b*_0_ = sin *q*_0_, *g*_0_ = cos *q*_0_, *c*_0_ = *p*_0_ sin *q*_0_, and *d*_0_ = *p*_0_ + cos *q*_0_.

The conservation of the Hamiltonian indicates $\frac{dH}{dt}=0$. In the present numerical simulation, if |Δ*H*| ≤ 10^−16^ (i.e., the smallest relative error in double-precision floating point arithmetic); it is regarded as unchanged. When *H* is a non-zero constant, $\left|\frac{\Delta H}{H}\right|\leq 10^{-16}$ is a criterion for a large Hamiltonian.

For instance, there is a Hamiltonian constant, *H* = −0.01, for Eq 2 when the numerical solutions of *p* and *x* are *p*_*N*_ and *x*_*N*_, respectively. The error of the Hamiltonian is

$$\Delta H=H_{N}-H=\left\{\frac{p_{N}{}^{2}}{2}+\frac{1}{2}\left(y_{N}{}^{2}-2y_{N}\right)\right\}-\left\{\frac{p^{2}}{2}+\frac{1}{2}\left(y^{2}-2y\right)\right\}.$$

Since

$$\left|\Delta H\right|\leq \left|\frac{p_{N}{}^{2}}{2}-\frac{p^{2}}{2}\right|+\frac{1}{2}\left|y_{N}{}^{2}-y^{2}\right|+\left|y_{N}-y\right|,$$

and

$$\left|\frac{p_{N}{}^{2}}{2}-\frac{p^{2}}{2}\right|+\frac{1}{2}\left|y_{N}{}^{2}-y^{2}\right|+\left|y_{N}-y\right|<10^{-16}$$

guarantees |Δ*H*| < 10^−16^. The numerical error at time *t* indicates that |*p*_*N*_ − *p*| ≤ *C*_1_*h*^*M*+1^ and |*y*_*N*_ − *y*| ≤ *C*_2_*h*^*M*+1^.

$$\left|\frac{p_{N}{}^{2}}{2}-\frac{p^{2}}{2}\right|\approx \left|p\left(p_{N}-p\right)\right|\leq \left|p\right|C_{1}h^{M+1}$$

where *C*_1_ and *C*_2_ are constants, and $\left|\frac{y_{N}{}^{2}}{2}-\frac{y^{2}}{2}\right|\approx \left|y\left(y_{N}-y\right)\right|\leq \left|y\right|C_{2}h^{M+1}$. Since |*p*| and |*y*| are bounded variables,

$$\left|\frac{p_{N}{}^{2}}{2}-\frac{p^{2}}{2}\right|+\frac{1}{2}\left|y_{N}{}^{2}-y^{2}\right|+\left|y_{N}-y\right|<Ch^{M+1},$$

where *C* is a constant that satisfies |*p*|*C*_1_ + |*y*|*C*_2_ + *C*_2_ ≤ *C*.

With a step-size of *h* = 0.01, a high enough order *M* is chosen to guarantee $\left|\frac{p_{N}{}^{2}}{2}-\frac{p^{2}}{2}\right|+\frac{1}{2}\left|y_{N}{}^{2}-y^{2}\right|+\left|y_{N}-y\right|<Ch^{M+1}<10^{-16}$. In practice, the order of *M* can be easily determined by several numerical runs without knowing the value of *C*. By using this high-order method, the structure-preserving solution of the original equation is obtained by numerical means. In fact, because it is very easy to increase *M* with the Taylor series method, we can make |Δ*H*| even smaller to meet Eq 2.

**Fig 1:**
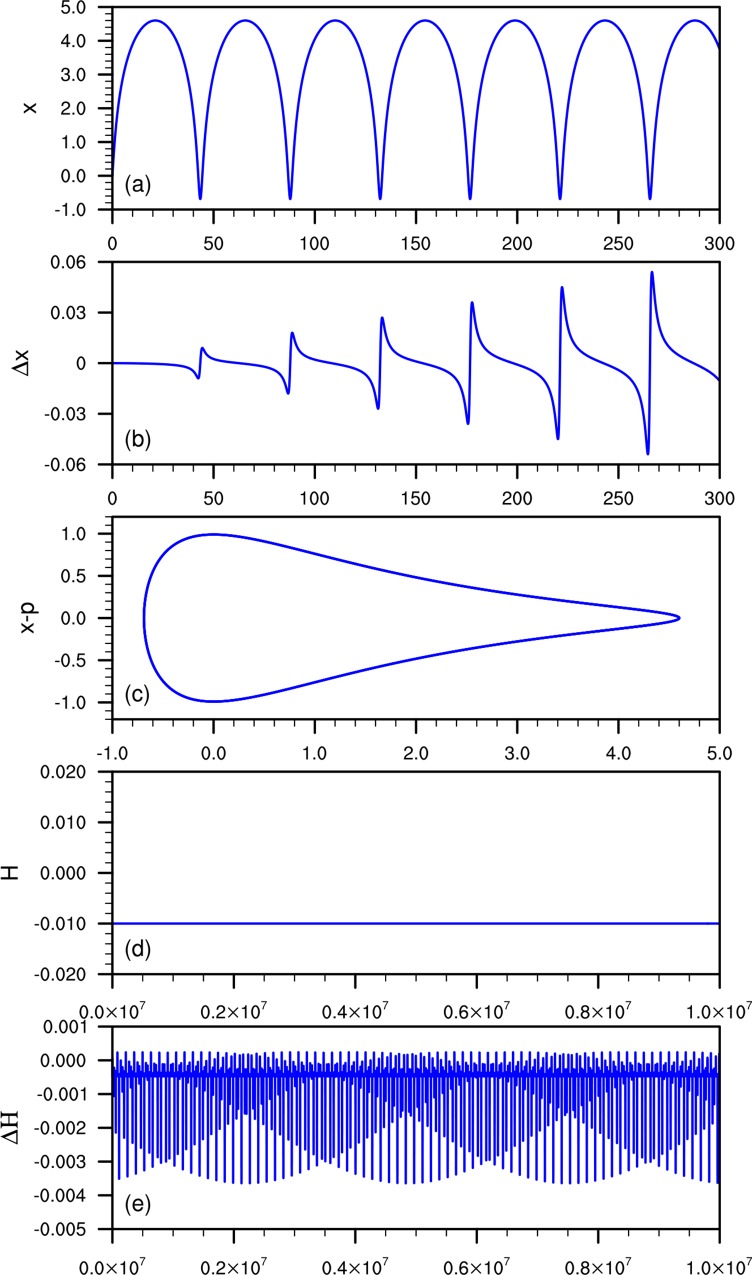
Illustrations of the direct simulation of Eq 2: (a) variable *x* by PMT; (b) error of *x* computed by the SE2 method; (c) structure of the *x*-*p* plane by PMT; (d) the Hamiltonian *H* by PMT to *T* = 10^7^; (e) error of the Hamiltonian *H* by the SE2 method. Panels (a–c) have step-size *h* = 0.01; (d–e) have step-size *h* = 0.1.

In the direct simulation of Eq 2 with *t* = 10^7^, a 20-order PMT scheme is used to achieve the simulation results. Fig 1 illustrations of the direct simulation of Eq 2. In Fig 1C, the PMT method is shown to predict the correct trajectory structure (*x*-*p* plane) along with a correct cycle of *x* (Fig 1A). During the entire computation time range, the Hamiltonian *H* approaches a constant (Fig 1D), while Fig 1E shows that *H* varies periodically and has larger errors when using the SE2 method. From Fig 1B we could found a more important issue is that the error in *x* increases as the simulation time increases. Thus, the position of *x* is not reliable at times longer than 10^5^ time units.

**Fig 2:**
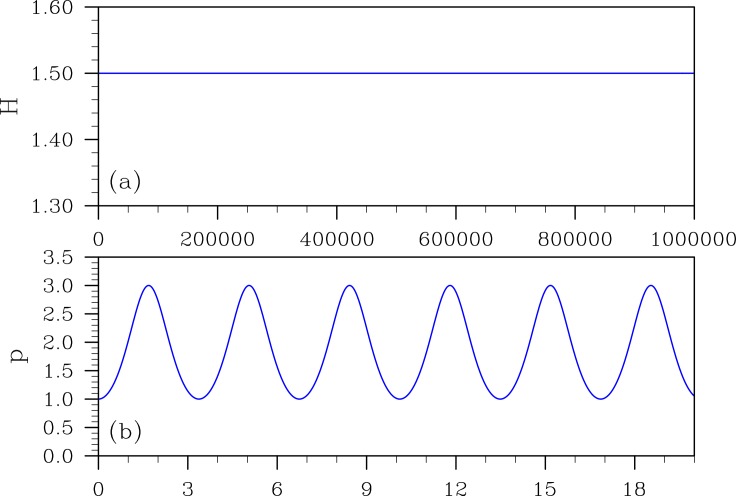
The direct simulation of Eq 5 by the 20-order PMT method, with a step-size of *h* = 0.01: (a) Hamiltonian *H*, (b) variable *p* versus time (the first 20 time units).

Fig 2 is the direct simulation of Eq 5 by the 20-order PMT method. Meanwhile, using the PMT method to solve the non-separable equation, the variable (Fig 2B) and the Hamiltonian *H* (Fig 2A) are simulated well. The Hamiltonian *H* remains to be a constant throughout the simulation in Fig 2A.

These results are all indicates that the PMT scheme can be used to simulate dynamical system with very high precision. Furthermore, the PMT scheme is a self-verifying scheme, as discussed in [7]. This verification scheme is a standard operation for PMT and CNS [12,15,16] numerical experiments.

The (Forward period analysis) FPA computation procedure is combined by two stages, one is the period finding and another is application of the founded period to do long term simulation. The FPA (and PMT scheme) are all suitable to solve the linear problem, but here we use a nonlinear system as objection to test the scheme’s performance. The more details of FPA are reported in the following section.

## Results

### FPA stage 1: The period discover of a Hamiltonian system

Establishing the phase trajectories of Hamiltonian systems is a basic requirement, which can be achieved by the symplectic method as well as PMT. However, the symplectic method only gives approximate numerical periods, with a precision proportional to the order of the method and step-size.

The key of numerical methods to identify the period of a dynamical system is to find out when the solutions return to the initial values (if the system is defined by *n*-th 1^rst^ order differential equation, the *n* variables must return to their initial values simultaneously). The integration time between the start point and the repeat point is thus approximately the period. Generally, the period obtained by numerical method in this way varies. The error between a variable (such as *x*) and its corresponding repeat point (defined as *E*_*x*_, for example *E*_*x*_ ≤ 10^−30^) indicates the uncertainty (Δ*t*) in estimating the period. Because *E*_*x*_ is small and $\frac{dx\left(t\right)}{dt}\approx \frac{E_{x}}{\Delta t}$, Δ*t* ⋅ *p* ≈ *E*_*x*_. The standard division of *p* is obtained through numerical experiments, $\sigma \left(p\right)=\sqrt{\bar{p^{2}}}$, as an averaged value of *p* such that Δ*t* ∼ *E*_*x*_ / *σ*(*p*). This formula suggests that the error bounds of a typical period have a magnitude of 10^−30^.

The forward period analysis (FPA) method is proposed to obtain the period for Eq 2. The first stage of FPA is a pre-computation to find a suitable residual interval. The computation starts with *y* = 1, $p=\sqrt{1-0.02}$ and $\dot{y}=-py=-\sqrt{1-0.02}$, and a time-step size of *h*_0_ = 0.01. At each step, the values of *y*_*k*_ and *y*_*k*−1_ are checked to find out the approximately first period. The first repeat position satisfies *y* ≈ 1 and $\dot{y}\approx -\sqrt{1-0.02}$, determined within the interval of [*T*_*l*_,*T*_*h*_], where *T*_*l*_ is the lower and *T*_*h*_ the higher bound. This interval is the time it takes for *y* to cross the base line *y* = 1 (from the *y* > 1 to the *y* < 1 direction, the steps that reach the lower bound are defined as *k*). The first period is now between *T*_*l*_ = *kh*_0_ and *T*_*h*_ = (*k* + 1)*h*_0_ time units, and thus the period *T* ≈ *T*_*l*_. The error of the period is about (*h*_0_ + Δ*t*) ≈ 10^−2^, and thus the precision of this forward period analysis method is mostly dependent on the last computation step-size *h*_*F*_ (in this stage, *h*_*F*_ = *h*_0_).

**Fig 3:**
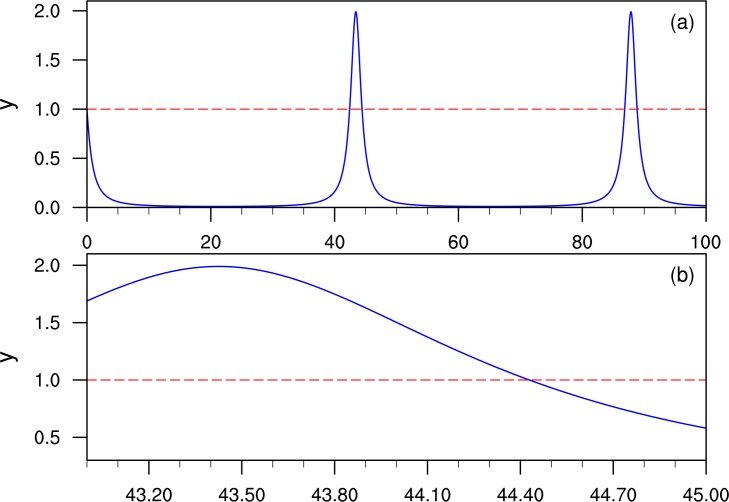
Demonstration of the FPA method to obtain the period for Eq 2: (a) the numerical result of variable *y* by the PMT method in the interval [0,100]; and (b) variable *y* in the enlarged interval of [43,45].

The second stage is the post-computation at the residual interval [*kh*_0_,(*k* + 1)*h*_0_] where *k* = Int(*t* / *T*) is a positive integer number. This interval can be separated into subintervals by the dichotomy method. Denoting the whole interval as before, [*T*_*l*_,*T*_*h*_], with *T*_*l*_ = *kh*_0_ and *T*_*h*_ = (*k* + 1)*h*_0_, a new step size *h*_*F*_ = *h*_*F*_ / 2 is chosen to separate the interval into [*T*_*l*_,*T*_*l*_ + *h*_*F*_] and [*T*_*l*_ + *h*_*F*_,*T*_*h*_]. If the value of *y* at *T*_*l*_ + *h*_*F*_ does not cross the base line, then *T*_*l*_ = *T*_*l*_ + *h*_*F*_; otherwise, *T*_*h*_ = *T*_*l*_ + *h*_*F*_ and then the operation is repeated in the new interval [*T*_*l*_,*T*_*h*_]. The dichotomy method maintains *h*_*F*_ smaller than the magnitude of *E*_*T*_ / *σ*(*p*), and thus total error of the period is dominated by Δ*T* (Δ*T* < *T*_*h*_ − *T*_*l*_ ≈ 10^−30^). The computation cost for the dichotomy method in the last interval is about 30log_2_ 10 ≈ 100 loops, while that in the pre-computation stage is about *T* / 0.01 loops. The value of *T* can be roughly estimated from Fig 3A (*T* is within a 45 time unit). The total loops for obtaining the period of Eq 2 are within 5000 loops.

The above procedures are also suitable for SE2 and other symplectic methods; if we choose the step-size (*h*) for SE2, the period with precision at the magnitude of *h*^2^ is obtained. Applying the dichotomy method at the last interval for SE2, the error at *T*_*l*_ and *T*_*h*_ must first be confirmed to be small enough, and this is not the superiority for SE2. Since a decreas in *h* greatly increases the computation time, if a more accurate period is required, for example Δ*T* = 10^−30^, *h* ≈ 10^−15^ must be set, and this requires 10^15^ loops to finish the computation.

In addition, applying self-verification requires reducing step-size to be about *h*/100 or less in SE2 to guarantee that the reference solution is more accurate than the solution in which step-size is *h*, and this requires 100 times more loops than the computation process. While self-verification of the PMT method only requires increasing the order *M* to a bigger value for example (*M*+10), this does not increase the number of computation loops of the verification process, but only the time cost per loop. The increasing time cost in one integration loop is insignificant when *M* is large (for example *M*>100). As a consequence, the PMT method is efficiently verified.

Fig 3 is the demonstration of the FPA method to obtain the period for Eq 2. After we obtain the roughly value of period T from Fig 3A. we then can obtain a more precise value from the enlarged time axis in Fig 3B. In Fig 3B the first stage of FPA is to determine the residual interval of Eq 2 as [44.42,44.43], i.e. *T*_*l*_ = 44.42 and *T*_*h*_ = 44.43. The FPA procedure in this interval, and the values of 30 and 100 significant digits, are listed in Table 1. To the author’s knowledge, this level of accuracy has never been reported.

**Table 1:** The period of a Morse system obtained by FPA with *M* = 200, *h* = 0.01, and the precision we use is 2000 bits.

Significant digits	*T*
30	44.4288293815836624701588099006
100	44.42882938158366247015880990060693698614621689375690223085395606956434793099473910575326934764765237

### FPA stage 2: The application of FPA in long-term simulations

Before demonstrating an ultra long-term simulation of Eq 2, a simple periodic dynamical system is analyzed to determine the most important parameters in the long-term computation. The simple dynamical system is defined by

$$\{\begin{array}{c} dx/dt=p\\ dp/dt=-x \end{array}$$ (6)

and the initial values are

$$\{\begin{array}{c} x\left(0\right)=0\\ p\left(0\right)=1 \end{array}.$$

The issue is how to obtain 16 significant digits of *x*(*t*) at *t* = 10^30^. Since the analytical solution of this equation is

$$\{\begin{array}{c} x\left(t\right)=\sin\left(t\right)\\ p\left(t\right)=\cos\left(t\right) \end{array},$$

the result should be *x*(10^30^) = sin(10^30^). As sin(*t* − 2*πk*) = sin(*t*), the result is given by sin(10^30^) = sin(10^30^ − 2*πk*), and $k=\text{Int}\left[\frac{10^{30}}{2\pi }\right]$, i.e., the integer part of $\frac{10^{30}}{2\pi }$. The precision of sin(10^30^) is dependent on the precision of the approximation to 2*π*. The reference value of *π* with 50 significant digits and the computed *k* are listed in Table 2, and the double-precision (16 significant digits) results are also compared. From Table 2, note that the *k* values corresponding to the two different precisions of *π* are different. The different *k*-values cause the residual of *t*, i.e., *T*_*r*_ = 10^30^ − 2*πk*, to be more uncertain. Therefore, precision to 16 significant digits for sin(10^30^) is not possible in a double-precision float platform.

**Table 2:** The values of *π* and *k*.

Significant digits	*π*	*k*
50	3.1415926535897932384626433832795028841971693993751	159154943091895335768883763372
16	3.141592653589793	159154943091895335768883763373

The above example indicates that the reliable long-term computation of a periodic system is dependent on the precision of the period; 2*π* can be regarded as the period in this example. The relative error bound, *ε*, is estimated for the period to limit the computation error at *t* to Δ*x* = 10^−16^. The true residual time can be written as

$$T_{r}=10^{30}-2\pi \text{Int}\left[\frac{10^{30}}{2\pi }\right],$$

and the residual time induced by numerical error is

$$T_{r}^{'}=10^{30}-2\pi \left(1+\varepsilon \right)\text{Int}\left[\frac{10^{30}}{2\pi \left(1+\varepsilon \right)}\right].$$

The first restriction of *ε* guarantees that $k=\text{Int}\left[\frac{10^{30}}{2\pi }\right]$ and $k=\text{Int}\left[\frac{10^{30}}{2\pi \left(1+\varepsilon \right)}\right]$ are the same. Under this situation, we have *T*_*r*_ = 10^30^ − 2*πk*, $T_{r}^{'}=10^{30}-2\pi \left(1+\varepsilon \right)k$ and the difference between *T*_*r*_ and $T_{r}^{'}$ is Δ*t* = 2*πkε*. The error bounds, *ε*, satisfying 2*πkε* < 10^−16^ will guarantee Δ*x* < 10^−16^. Since 0 < 10^30^ − 2*πk* < 2*π*, 2*πk* ≈ 10^30^ and *ε* < 10^−16^/2*πk* = 10^−46^ is the relative error bound of 2*π*. The 50 significant digits of *π* satisfy this error bound. Thus, *T*_*r*_ = 3.231831977487846 and sin(10^30^) = −0.090116901912138058.

The analysis of error bounds for a general periodic dynamical system is the same as the example by changing the period from 2*π* to *T*. Thus, *ε* < *E*(*t*)/*t* is the period error bound, where *t* is the simulation time and *E*(*t*) the required relative error bound for the output. The fast computation of sin(10^30^) is a benefit from the pre-known of precise value of *π*. However, for a general periodic dynamical system such as Eq 2 there is no such pre-knowledge for the period *T*, hence FPA is proposed to obtain the precise *T* first.

Long-term simulation by FPA is achieved by dividing the long-term (*t* ≫ *T*) computation into two parts: one is the detection of period of a cycle; the other is the simulation of the residual time, equal to *t* − *kT*. Because the computation error of the symplectic method generally propagates linearly with time [17,18], such that an increase of *t* times requires a *t*^1/*M*^ times smaller step-size to control the error, where *M* denotes the order of the symplectic method. The computation complexity for time *t* in unit loops is *O*(*t*^1+1/*M*^). However, applying the FPA procedure with PMT helps to reduce the computation from *O*(*t*^1+1/*M*^) to *O*(*T* / *h*_0_) in the first stage, and to *O*(ln *t*) in the second stage, i.e., *O*(ln *t* + *T* / *h*_0_).

As illustrated in Table 3, the long-term computation of a dynamical system uses at least *O*(*t*) loops without FPA, but with FPA this is cut to *O*(ln *t* + *T* / *h*_0_) at most. This improvement greatly decreases the CPU time cost and makes many unsolvable long-term problems be reliably solvable.

**Table 3:** The loops used in the different schemes.

Scheme	Direct integration	Applying FPA
Symplectic	*O*(*t*^1+1/*M*^)	*O*(ln *t* + *Tt*^1/*M*^)
PMT	*O*(*t* / *h*_0_)	*O*(ln *t* + *T* / *h*_0_)

Given the period obtained by FPA (see Table 1), the variable values for the Morse system will be computed quickly. The results of selected times from (10 to 10^60^) are listed in Table 4, with the corresponding values obtained by direct integration. The results of direct integrations with FPA and PMT agree well. The results of the SE2 method have errors from the early integration stage, and the results beyond 10^5^ are incorrect. It took about one day to obtain the result at *t* = 10^7^ by direct integration, so obtaining a result at 10^60^ is a seemingly impossible task for direct integration, but with the help of FPA, reliable results can be obtained.

**Table 4:** The variable *p* obtained by FPA and direct simulation for a Morse system.

t	Direct integration with SE2	Direct integration with PMT	By FPA with PMT
0	0.989949493661166	0.989949493661166	0.989949493661166
10	0.142033683767425	0.142049967327890	0.142049967327890
10^2^	0.120638240019144	0.120968440888269	0.120968440888269
10^3^	−0.015582896828410	−0.013519353495639	−0.013519353495639
10^4^	0.275552330918520	0.406695207104251	0.406695207104251
10^5^	0.120371703265026	−0.209442226126745	−0.209442226126745
10^6^	−0.017153824006555	−0.575071021680786	−0.575071021680786
10^7^	0.214900072283681	0.406850634713920	0.406850634713920
10^8^	-	-	−0.208888390114776
10^9^	-	-	−0.548460247715134
10^10^	-	-	0.632436322896067
10^20^	-	-	−0.459751580833174
10^30^	-	-	0.203324673559885
10^40^	-	-	0.240998707662065
10^50^	-	-	0.544244466975686
10^60^	-	-	−0.839008449972302

Another classical dynamical system is the mathematical pendulum. The Hamiltonian of a pendulum system is $H=\frac{1}{2}p^{2}-\cos q$, and the dynamical equation is

$$\left\{ \begin{array}{l} \dot{q}=p\\ \dot{p}=-\sin q \end{array}\right.$$ (7)

with initial values

$$\{\begin{array}{c} q\left(0\right)=0\\ p\left(0\right)=1 \end{array}.$$

The period of this system approaches to 2*π*, while the initial momentum *p* → 0 [17]. Table 5 lists the period corresponding to the initial condition, *q* = 0, with different momenta, *p*. All periods are accurate to 50 significant digits. As illustrated in Table 5, the period approaches 2*π* when *p* decreases from 1 to 10^−30^. This experiment again proves the correctness of FPA.

**Table 5:** The period of a mathematical pendulum system obtained by FPA with *M* = 200, *h* = 0.01, and the precision we use is 2000 bits.

*P*	*T* (50 significant digits)
1	6.7430014192503841714848146311963079580032035765643
10^−1^	6.2871178299331781141446745665180361610970124356918
10^−2^	6.2832245776399990205430348375448192284997546476674
10^−3^	6.2831856998787233989673928392330685974085610706684
10^−4^	6.2831853111065772994348591606129983671149348353934
10^−5^	6.2831853072188563850957114151234372289079555651010
10^−10^	6.2831853071795864769292137573759930099424226253101
10^−20^	6.2831853071795864769252867665590057683943780686583
10^−30^	6.2831853071795864769252867665590057683943387987502
2*π*	6.2831853071795864769252867665590057683943387987502

It is very fast applying FPA to obtain a period for these demonstration systems, and the computation can be finished within 1 minute on a Linux system with an Intel Xeon 2.5 Ghz CPU. The long-time scope solutions can be obtained within 1 minute by FPA.

## Discussion and Conclusion

Using the FPA, we obtain the periods of some classical Hamiltonian systems, with the accuracy of 100 significant digits. We also confirm that reliable solutions within the time range *t* ∈ [0,10^60^] can be obtained. To the best of the author’s knowledge, this accuracy of time period for long-term solutions of Hamiltonian systems has not been reported before. The FPA method provides a powerful tool to gain time-effective ultra long-term reliable solutions of periodic systems.

The FPA procedure works well in conjunction with the traditional symplectic method and the PMT method. Generally, symplectic methods with different orders require different subroutines to conduct the computation. In contrast, it is relatively easy to change the order of the Taylor series method, so that it provides the flexibility to carry out simulations with different orders of accuracy for one system. The PMT method reasonably simulates the Morse system for *t* ∈ [0,10^7^], but for much longer times simulation it is hard without FPA. This is demonstrated using a simple example. For more complex systems, higher order PMT approaches can be used. Indeed, details of an example for application of a high-order PMT method in direct simulationg of the Henon–Heiles system is referred to Liao [15].

The Taylor series method has a good convergence property when the order is high enough [9]. This feature can enlarge the step-size *h* to 0.01 for Eq 2, and increase the simulation speed. The result obtained by the Taylor series method not only maintains Δ*H* ≃ 0, but also reduces numerical errors. The PMT method is not a structure-preserving method, but it can preserve the structure well by numerical means. Consequently, it can be used as an alternative of symplectic methods for the computation of simple Hamiltonian systems. Moreover, PMT can be applied to some non-separable nonlinear Hamiltonian systems as well as separable ones, and even to non-Hamiltonian chaotic systems.

The essence of applying FPA to long-term computation is divided into two parts. One is the period detection of a unit cycle. The other is the computation of residue time equal to *t* − *kT*. This procedure helps to reduce the computation time for the long-term reliable simulation from *O*(*t*^1+1/*M*^) to *O*(ln *t* + *T* / *h*_0_). The FPA procedure improves not only the PMT method, but also facilitates the traditional symplectic method. The main problem of the symplectic method is that if the order *M* is not large enough (for example *M*<10) it still requires many computation loops for *t* = 10^60^ –about *O*(60 ln 10 + *T*10^60/*M*^) loops. For a medium-term time period, such as the ~10^9^ orbits of the Earth–solar system, the solution is required at a *t* = 10^17^ seconds magnitude. In this case, the symplectic method should work as well as FPA.

Recently, Barrio et. al[19] provided a shooting-periodical method which is a faster algorithm to obtain solutions in [0,10000] for a pre-obtained initial value for Lorenz system. The author notices that the long-term database obtained by Barrio is for the special initial values, while the method here is for any general initial values. It is well known that some Hamiltonian systems such as bounded Kepler system always have periodical orbits. Thus, in this case no shooting methods are needed to obtain the property initial values. While other Hamiltonian systems such as Arenstorf orbits[20] problems which have more freedom initial dimensions perhaps need such shooting methods to obtain precise initial values.

In this study, the author focuses on the long term simulation of periodic Hamiltonian systems, and not considers the spatial effect. When we count the spatial dimensional the ODE(ordinary differential equation) will change to PDE(partial differential equation), some of this PDE system still have periodic properties. For instant, the barotropic vorticity equation on the sphere[21]. While other systems may not have periodic for instant the Allee effect[22] in population dynamics. Two difficulties may occur when apply FPA to deal with these systems. Firstly, the determination of periodic of PDE system is more complicate then the ODE. Secondly, their may have no high enough integration scheme to do the integration within one periodic, and thus cause the founded periodic not very accurate. Nevertheless, as Sun et,al. pointed that the spatial dynamic pattern describe by PDE is ubiquitous in nature[23], thus improve FPA to do computation of such PDE dynamical system in an important work in the future study.
